# Supplementary material for: Study of the Antimicrobial Activity of the Human Peptide SQQ30 against Pathogenic Bacteria
Source: Antibiotics (Basel). 2024 Feb 1;13(2):145. doi: 10.3390/antibiotics13020145 (PMC10886087; doi:10.3390/antibiotics13020145)
Supplement: Supplementary file 1 [file antibiotics-13-00145-s001.zip › antibiotics-2813433-supplementary.pdf]

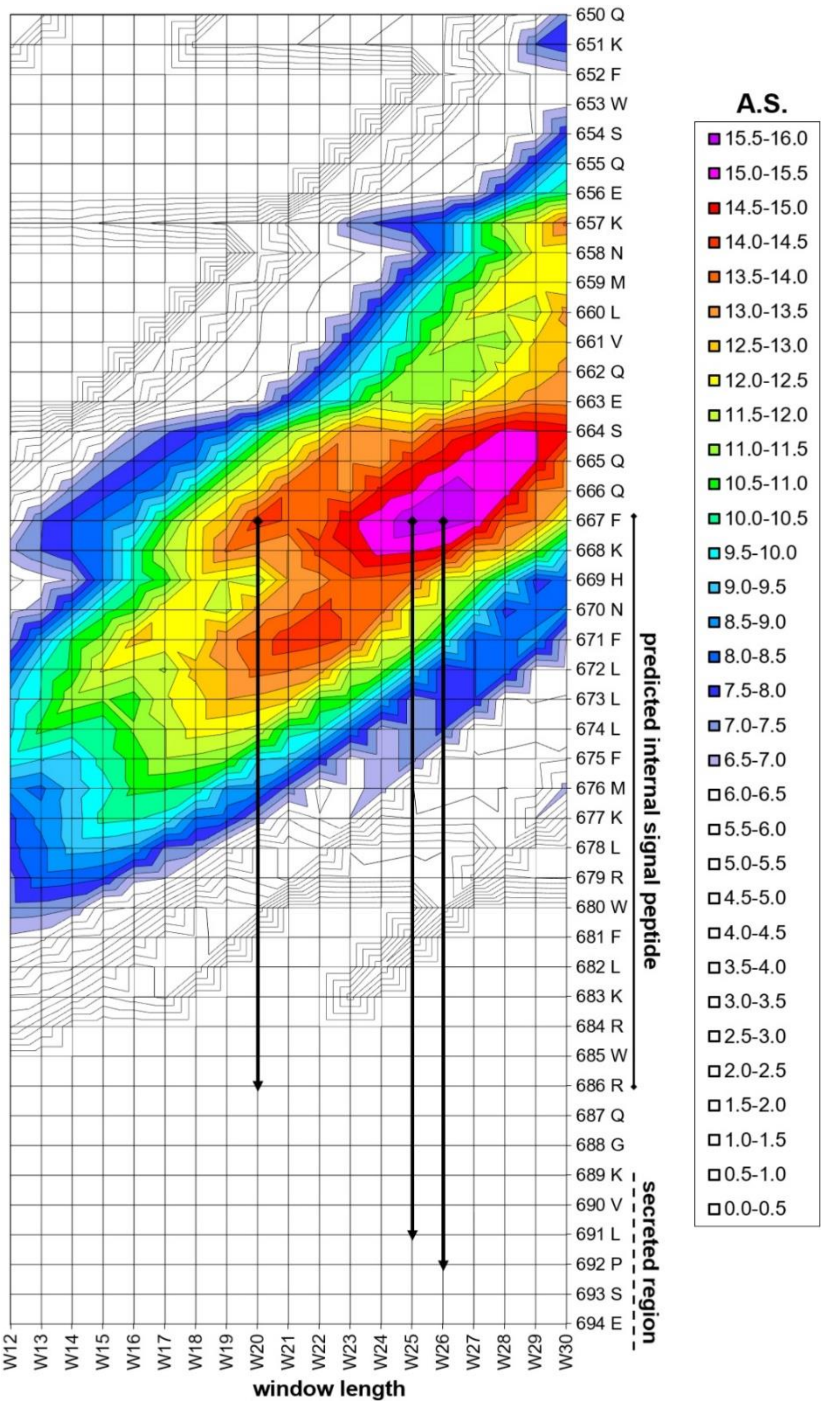

Figure S1. Sliding window analysis of the region 650-694 of the human protein SOGA1 (UniProt code O94964). The sequence of the protein is shown on the vertical axis (UniProt numbering). The left arrow highlights a local A.S. maximum corresponding exactly to the predicted signal peptide (Phe667-R686). The two arrows on the left highlight the highest A.S. values corresponding to peptides 667-691 and 667-692. The sliding window analysis was performed using the hydrophobicity scale “Parker-Arg0” and the strain-dependent parameters calculated for strain S. aureus C623 [Pane et al., J. Theor. Biol. 2017, 419, 254–265].

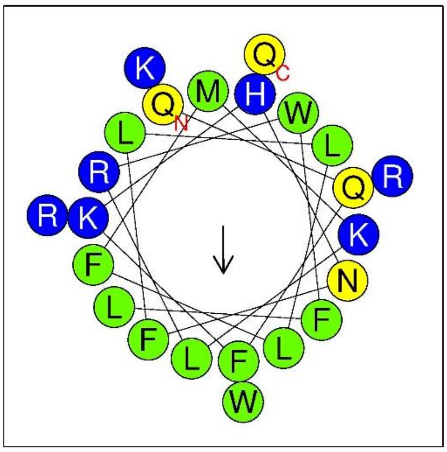

Figure S2. Helical wheel projection of the region Gln665-Gln687 of SOGA1 (QQFKHNFLLLFMKLRWFLKRWRQ) generated by the HeliQuest server (https://heliquest.ipmc.cnrs.fr/). Color code; blue, basic residues; yellow, polar uncharged residues; green, hydrophobic residues. The harrow indicates the hydrophobic moment of the helix (μ = 0.343; HeliQuest prediction).

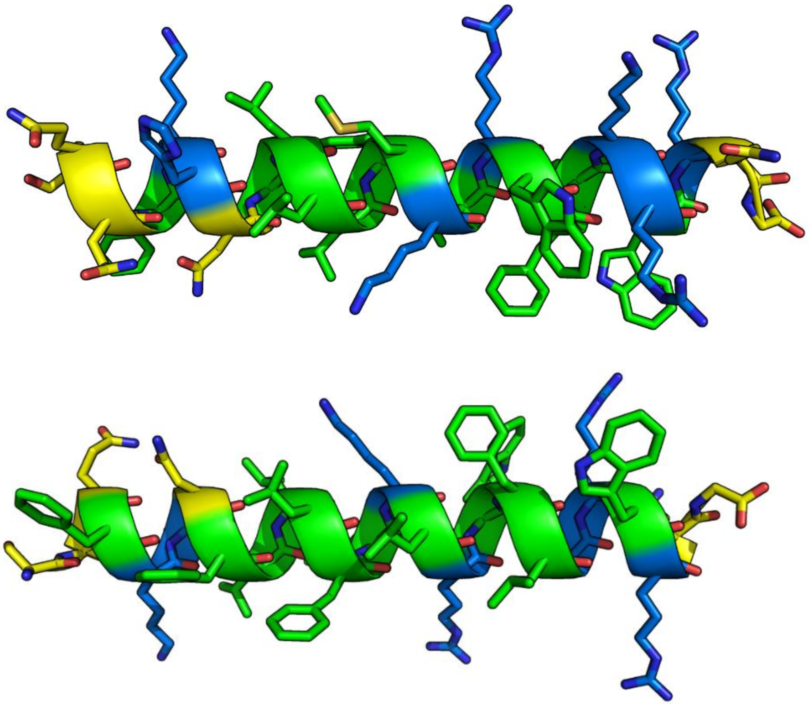

Figure S3. Alpha-helical model of the region 664-688 of SOGA1 seen from the hydrophilic face (upper panel) and from the hydrophobic face (lower panel). Residues are colored by atom type: nitrogen, dark blue; oxygen, red; sulfur, dark yellow; carbon, green (hydrophobic residues), blue (basic residues), yellow (polar/uncharged residues). The model was generated using PyMol (https://pymol.org/2/) and energy minimized using Swiss PDB viewer (https://spdbv.unil.ch/). The pictures were generated using PyMol.

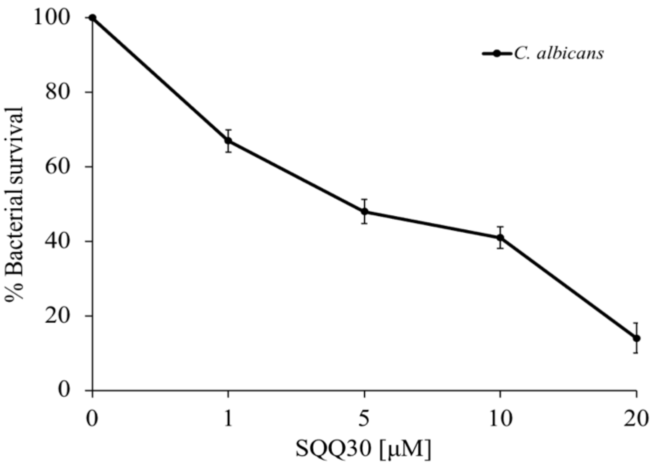

Figure S4. Antifungal activity of the peptide tested against *C. albicans*. The assays were performed for three independent experiments. Standard deviations are always less than 10%.

Table S1. Evaluation of the minimum concentration values inhibiting fungal growth (MIC_{100}) of SQQ30 against *C. albicans* present in the oral cavity. The values were obtained from a minimum of three independent experiments.

**Strains**	**MIC [μM]**
C. albicans	40

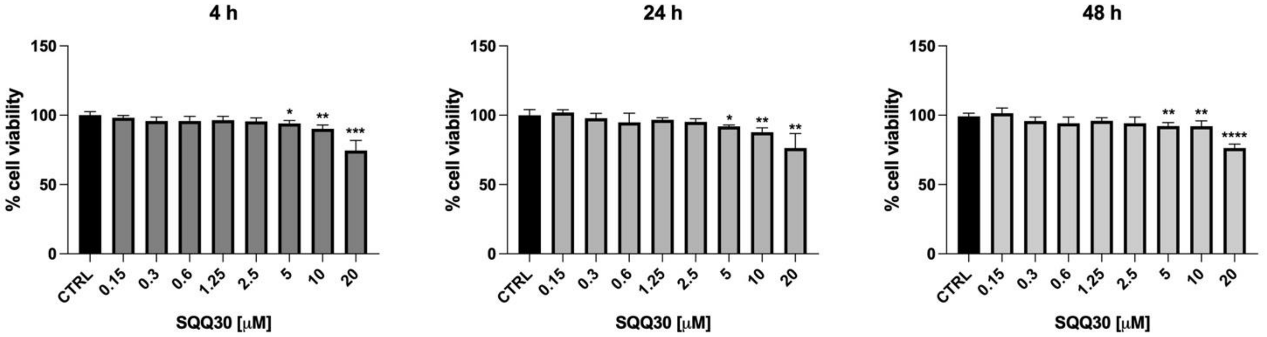

Figure S5. Viability of Raw 264.7 murine macrophages cells treated with SQQ30. Increasing concentrations of SQQ30 (from 0.15 μM to 20 μM) were administrated to cells for 4, 24 and 48 hours. Cell viability was determined by the MTT assay. Statistical analysis was carried out by GraphPad Prism using Student's t-test (* $p < 0.05$, ** $p < 0.01$, *** $p < 0.001$ and **** $p < 0.0001$) versus untreated cells.

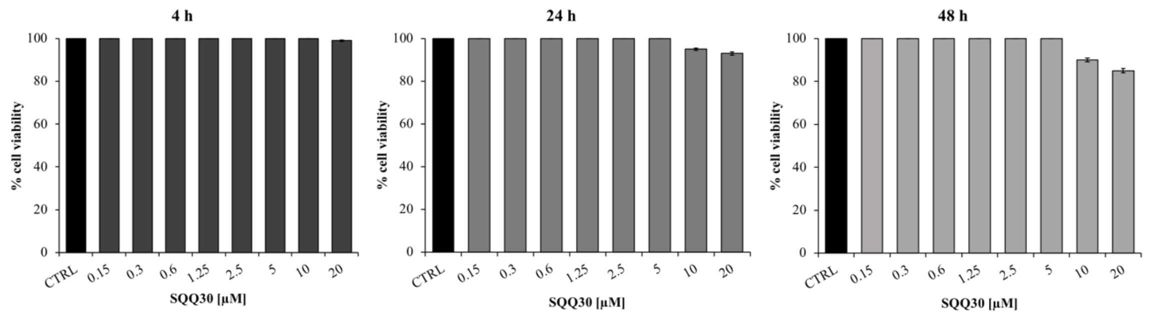

Figure S6. Viability of HaCat cells treated with SQQ30. Increasing concentrations of SQQ30 (from 0.15 µM to 20 µM) were administrated to cells for 4, 24 and 48 hours. Cell viability was determined by the MTT assay. Data represent the means of three experiments ± S.D.
